# Monoamine Oxidase Is Overactivated in Left and Right Ventricles from Ischemic Hearts: An Intriguing Therapeutic Target

**DOI:** 10.1155/2016/4375418

**Published:** 2016-12-01

**Authors:** Maria Elena Manni, Stefania Rigacci, Elisabetta Borchi, Valentina Bargelli, Caterina Miceli, Carla Giordano, Laura Raimondi, Chiara Nediani

**Affiliations:** ^1^Department of Neurosciences, Psychology, Drug Research and Child Health (NEUROFARBA), Center of Molecular Medicine (CIMMBA), University of Florence, Florence, Italy; ^2^Department of Experimental and Clinical Biomedical Sciences, University of Florence, Florence, Italy; ^3^Department of Radiological, Oncological and Pathological Sciences, Sapienza University of Rome, Rome, Italy

## Abstract

Growing evidence indicates that reactive oxygen species (ROS) may play a key role in human heart failure (HF). Monoamine oxidase (MAO) is emerging as a major ROS source in several cardiomyopathies. However, little is known about MAO activity in human failing heart and its relationship with redox imbalance. Therefore, we measured MAO activity in the left (LV) and in the right (RV) ventricle of human nonfailing (NF) and in end-stage ischemic (IHD) and nonischemic failing hearts. We found that both MAO isoforms (MAO-A/B) significantly increased in terms of activity and expression levels only in IHD ventricles. Catalase and aldehyde dehydrogenase-2 activities (ALDH-2), both implicated in MAO-catalyzed catecholamine catabolism, were significantly elevated in the failing LV, whereas, in the RV, statistical significance was observed only for ALDH-2. Oxidative stress markers levels were significantly increased only in the failing RV. Actin oxidation was significantly elevated in both failing ventricles and related to MAO-A activity and to functional parameters. These data suggest a close association between MAO-A-dependent ROS generation, actin oxidation, and ventricular dysfunction. This latter finding points to a possible pathogenic role of MAO-A in human myocardial failure supporting the idea that MAO-A could be a new therapeutic target in HF.

## 1. Introduction

Heart failure (HF) is a cardiovascular syndrome with high morbidity and mortality characterized by complex pathophysiology. The failing heart is the final step of different cardiomyopathies. The altered myocardial phenotype and metabolism lead to a progressive loss of left ventricular efficiency. Among the mechanisms involved in HF etiopathogenesis, a growing body of evidence suggests that reactive oxygen species (ROS) and oxidative stress may play a key role in both the initiation (myocardial remodelling) and the progression towards overt HF [1]. A number of intracellular ROS sources have been identified in animal and human models of cardiac diseases, including NADPH oxidase (NOX), xanthine oxidase, and nitric oxide synthase activities. The mitochondrial respiratory chain is certainly the best-characterized site for ROS generation in the cell. Recently, an additional mitochondrial protein, monoamine oxidase (MAO), is emerging as a major ROS source with potential pathophysiological relevance [2, 3]. MAO is an ubiquitously expressed FAD-dependent enzyme localized at the outer mitochondrial membrane that exists in two isoforms, MAO-A and B, with peculiar tissue distribution, substrate selectivity, and inhibitor specificity [4]. MAO-A substrates include noradrenaline (NE) and serotonin (5-HT), while phenylethylamine is a specific substrate of MAO-B. Oxidative deamination of MAO substrates that produces hydrogen peroxide and the corresponding aldehyde is selectively prevented by propargylamines (clorgyline and deprenyl) [5]. Many studies have extensively highlighted the role of MAO activity in the central nervous system [6–8]; however, its relevance in other organs, including the heart, has been investigated only recently. Increasing evidence suggests that, in rodent cardiomyocytes, MAO activity may contribute to NE- and 5-HT-induced hypertrophy, to apoptosis, and to the stimulation of cell glucose uptake by hydrogen peroxide generation [9–13]. These hydrogen peroxide-mediated effects are particularly relevant since they unmask a new receptor-independent roles of catecholamines and 5-HT [14, 15], prompting the investigation on the possible role of MAO in cardiac diseases, including HF. Indeed, recent studies in animal models suggest that MAO-A/B activation plays a crucial role in the progression from heart hypertrophy towards failure, establishing a clear association between MAO-induced ROS production, mitochondrial dysfunction, and cardiac failure, leading to propose MAO as a new promising therapeutic target in chronic diseases [3, 9, 15–20]. Despite the clinical relevance of these findings and the potential indication of MAO inhibitors for the treatment of heart failure, little is known about MAO activity in human failing heart and its relationship with redox imbalance. This latter is a well-accepted, although not the sole, determinant of the biochemical, metabolic, and functional derangement leading to cardiac maladaptive response [1]. In the context of human HF, MAO-generated ROS may be counterbalanced by catalase (CAT), the antioxidant enzyme that catalyzes the conversion of hydrogen peroxide into water and oxygen [21], as well as by glutathione and thioredoxin-2 antioxidant systems [21, 22]. Moreover, other cytotoxic products of MAO activity and of lipid peroxidation such as aldehydes may be scavenged by aldehyde dehydrogenase-2 (ALDH-2), the mitochondrial isoform of ALDH. Aldehyde dehydrogenases (ALDHs) are a superfamily of NAD(P)^+^-dependent enzymes that oxidize aldehydes to their corresponding carboxylic acids. In the myocardium, ALDH-2 plays an important role in the removal of toxic aldehydes and protects the heart against oxidative stress-injury [23]. Many studies have reported that ALDH2 plays a protective role in models of cardiovascular disease [23–28], whereas the lack or inhibition of ALDH2 exacerbates the consequences of MAO-A [29] or MAO-B [20] activation because of the accumulation of toxic aldehydes. The latter findings have been recently obtained in an experimental murine model of HF via pressure overload, in which Kaludercic et al. [20] reported that aldehydes, generated by amine catabolism via MAO-B, play a major role in MAO-mediated mitochondrial dysfunction that, in turn, leads to myocardial failure.

The present study was aimed at the following: (i) investigating the activity of MAO isoforms (MAO-A and B) in the left (LV) and in the right (RV) ventricle of nonfailing (NF) and end-stage human failing hearts from ischemic (IHD) and nonischemic (non-IHD) etiology; (ii) measuring the activity of the enzymes implicated in MAO-catalyzed catecholamine catabolism, namely, CAT and ALDH-2; (iii) assessing the occurrence of oxidative stress markers, malondialdehyde (MDA), and protein carbonyls; (iv) evaluating the oxidation of sarcomeric myofibrillar proteins that may be critical for the development of contractile impairment; (v) establishing a possible correlation between the activity of MAO-A, the isoform indicated as a possible responsible contributing to maladaptive heart remodelling and dysfunction, and functional parameters that are indexes of LV and RV impairment [30–32].

## 2. Materials and Methods

### 2.1. Tissue Origin and Preparation

The study conformed to the Declaration of Helsinki and institutional ethical regulations. Explanted failing hearts were obtained from patients undergoing transplantation for end-stage HF secondary to ischemic (IHD; *n* = 13) and non-IHD (non-IHD; *n* = 8) diseases, including idiopathic dilated and valvular cardiomyopathy. Nonfailing (NF; *n* = 7) donor hearts, unsuitable for transplantation for technical reasons, (i.e., size incompatibility between donor and recipient) or for past medical history of the donor (i.e., neoplasia), were used as controls. Clinical characteristics of the three groups are shown in Table 1. Immediately after explant, myocardial tissue samples were snap-frozen in liquid nitrogen-chilled isopentane for protein determination, RNA analysis, and biochemical assays. All tissues were stored at −80°C. Sections stained with hematoxylin-eosin and Masson Trichrome stain were obtained from each sample for morphological examination prior to each experiment. Histological slides were observed under light microscopy. Myocyte hypertrophy was a common finding in all failing hearts, associated with variable degrees of interstitial fibrosis, graded from mild (+1) to severe (+3) on a semiquantitative basis. According to the results of the histologic examination, myocardial samples with minimal amounts of fibrosis and devoid of inflammatory infiltrates were selected for molecular and biochemical studies. In addition, mRNA expression for atrial natriuretic factor, *α*- and *β*-myosin heavy chain isoforms, molecular markers of cardiac hypertrophy were evaluated quantitatively on both NF and failing hearts by RT-PCR using the Platinum SYBR Green qPCR Super Mix-UDG (Invitrogen).

### 2.2. MAO Activity

100–130 mg of tissue, from failing and NF LV and RV, was minced in liquid nitrogen and then homogenized by Ultra-Turrax in 0.1 M phosphate buffer (PBS) pH 7.8 containing 0.25 M sucrose. Then, the samples were centrifuged at 1000 ×g for 10 min to remove cell debris and nuclei. Protein content was evaluated by the BCA method (Pierce Scientific Rockford, IL61101, USA).

MAO activity was radiochemically assayed as previously described [33] using [^14^C]- 5-HT (1.0 *μ*Ci/mL; 100 *μ*M; Amersham Biosciences, UK) or [^14^C]-benzylamine (1.0 *μ*Ci/mL; 100 *μ*M; Amersham Biosciences, UK) as substrates for MAO-A and B, respectively. In particular, 40 *μ*L of tissue homogenates was preincubated in 100 *μ*L of PBS, pH 7.8, at 37°C for 30 min in the absence or in the presence of the MAO inhibitors pargyline (100 *μ*M; Sigma-Aldrich, St. Louis, MO, USA) or semicarbazide (1.0 mM; Sigma-Aldrich, St. Louis, MO, USA) and of semicarbazide-sensitive amino oxidases, respectively. Then, the labeled substrates were added to the enzyme preparations for 30 min at 37°C. The reactions were stopped by the addition of 20 *μ*L 3.0 N HCl. The aldehyde produced by enzyme reaction was extracted in ethyle acetate (300 *μ*L) and the organic phase was separated by brief spinning (1000 ×g for 5 min); then an aliquot (150 *μ*L) of the sample was counted for radioactivity in a *β*-counter. MAO activity was referred to as the radioactivity recovered in the organic phase corrected for the percentage of nonmetabolized substrate extracted in the organic phase. The results are expressed as nmol/mg of proteins/30 min.

### 2.3. Western Blot Determination of MAO Expression

Tissue samples were homogenized in ice-cold PBS, pH 7.4, supplemented with 1.0 mM PMSF, 10 *μ*M leupeptin, 10 *μ*M aprotinin 1.0 mM sodium orthovanadate, 1.0 mM sodium fluoride, and 1.0 mM sodium pyrophosphate using a Dounce tissue grinder, followed by two rounds of sonication (10 s each), and clarified at 1000 ×g for 10 min at 4°C. The supernatant was diluted in Laemmli sample buffer without bromophenol blue and 2-mercaptoethanol, boiled for 3 min, and centrifuged at 12.000 ×g for 10 min. Protein concentration in the cleared lysate was determined using the BCA method; equal protein amounts were separated on 12% SDS-PAGE and transferred to an Immobilon®-P membrane (Millipore Corporation, MA, USA). After blocking with 5.0% (w/v) BSA in 0.1% (v/v) PBS-Tween-20, the membrane was incubated overnight at 4°C with goat anti-MAO-B (1 : 1000, C-17) or rabbit polyclonal anti-MAO-A (1 : 1000, H-70) antibodies. Following 1.0 h incubation with donkey anti-goat (1 : 10 000, sc-2020) or goat anti-rabbit (1 : 10 000, sc-2004) secondary antibodies, the immunoreactive bands were detected by the chemiluminescent Westar Supernova substrate (Cyanagen, Italy) and quantified by densitometric analysis using a ChemiDoc system and the Quantity One software (Bio-RAD Laboratories, Italy). Following membrane stripping, mouse anti-*β*-actin (1 : 1000, sc-81178) and goat anti-mouse (1 : 10.000, sc-2062) were used as a reference for equal protein loading. All primary and secondary antibodies were from Santa Cruz Biotech (Santa Cruz, CA, USA).

### 2.4. CAT and ALDH-2 Activity Assay

CAT activity was measured spectrophotometrically in LV and RV homogenates as previously described [34] and expressed as nmol/min/mg protein.

ALDH-2 activity was assayed in the pellets obtained after centrifugation at 15,000 ×g for 30 min at 4°C of cardiac homogenates of LV and RV specimens; ALDH-2 activity was measured spectrophotometrically following the reduction of NAD^+^ at 340 nm after addition of benzaldehyde (400 *μ*M; Sigma-Aldrich, St. Louis, MO, USA) [35] and expressed as nmol/min/mg protein.

### 2.5. Protein Carbonyls and MDA Assay

Carbonyl residues were determined by the method of Correa-Salde and Albesa [36]. Cardiac homogenates (50 *μ*L) were treated for 1 h with 150 *μ*L of 0.1% 2–4 dinitrophenylhydrazine (DNPH) (Sigma-Aldrich, St. Louis, MO, USA) in 2.0 M HCl and precipitated with 10% trichloroacetic acid before centrifugation for 5 min at 10,000 ×g. The pellets were extracted three times with 500 *μ*L of an ethanol/ethyl acetate mixture (1 : 1) and then dissolved in 1.0 mL of 6.0 M guanidine-HCl (Sigma-Aldrich, St. Louis, MO, USA) in 20 mM PBS, pH 7.5. The solutions were incubated at 37°C for 30 min and the insoluble debris was removed by centrifugation. Sample absorbance was measured at 370 nm.

The MDA content, as a marker of lipid peroxidation, was evaluated with the specific “Bioxytech LPO-586” kit (Oxis International Inc), according to the manufacturer instructions.

### 2.6. Determination of Oxidized Proteins

The determination of oxidized proteins was performed using the OxyBlot™ Protein Oxidation Detection Kit (Millipore Corporation, MA, USA) according to the manufacturer instructions, with minor modifications. Tissue homogenates were obtained as for western blot analysis but the lysis buffer was supplemented with 1.0% (v/v) 2-mercaptoethanol to prevent protein oxidation after cell lysis. Following the 1000 ×g clarification step, protein concentration was determined using the Bradford reagent and 5 *μ*L of each sample containing 40 *μ*g of proteins was denatured by adding 5.0 *μ*L of 12% SDS and by sample incubation for 5 min at 90°C. Carbonyl groups introduced into protein side chains following oxidation were derivatized to 2,4-dinitrophenyl- (DNP-) hydrazone (DNPH) by reaction with DNPH for 15 min at RT. After neutralization, the samples were centrifuged at 12,000 ×g for 2 min, separated by 12% SDS-PAGE, and transferred to an Immobilon-P membrane (Millipore Corporation, MA, USA). Western blot was performed using a primary antibody specific for the DNP moiety of the proteins and a secondary goat anti-rabbit IgG, as indicated in the kit protocol. The immunoreactive bands were detected as described for the western blot determination of MAO. Antibody specificity for carbonylated proteins was preliminarily verified by running samples incubated with the provided Derivatization-Control solution, instead of with the DNPH solution. The identification of carbonylated proteins was performed by stripping and reprobing the membrane with mouse anti-tropomyosin (CH1) (1 : 1000, sc-58868) or anti-*α*-actin (5C5) (1 : 1000, sc-58670) antibodies (Santa Cruz Biotech, Santa Cruz, CA, USA).

### 2.7. Statistical Analysis

All values were expressed as mean ± SEM. Comparisons were performed using the Student *t*-test and One-Way ANOVA followed by Dunnett's* post hoc* test when indicated. Correlation coefficient *r* was obtained using a linear (Pearson) correlation test (GraphPadPrism 5). Results were considered to be significant at *p* < 0.05.

## 3. Results

### 3.1. Total MAO (A + B) Activity in NF and Failing Ventricles

**Figure 1:**
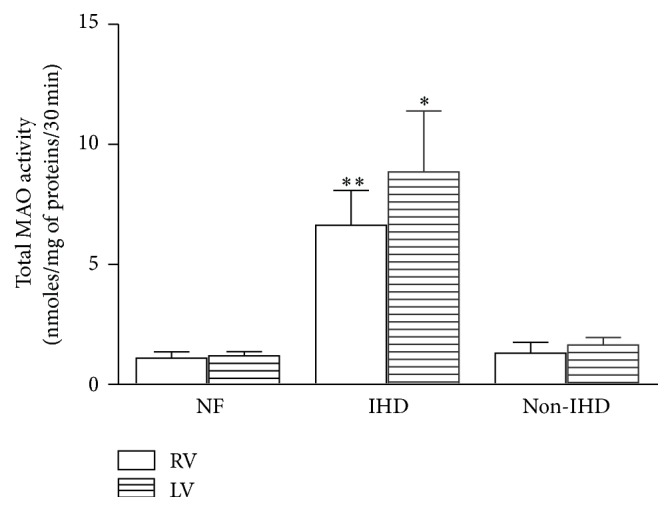
Total MAO activity in RV and LV of nonfailing (NF) and failing hearts secondary to ischemic (IHD) and nonischemic (non-IHD) diseases. Data are expressed as mean ± SEM. Comparison is performed using ANOVA followed by Dunnett's* post hoc* test. ^*∗∗*^ *p* < 0.01 IHD RV* versus* NF RV; ^*∗*^ *p* < 0.05 IHD LV* versus* NF LV. *n* = 7 NF; *n* = 13 IHD; *n* = 8 non-IHD.

Total MAO activity, as a sum of MAO-A and MAO-B activity, was measured in LV and RV of NF and failing samples from ischemic and nonischemic hearts. To exclude that age was a possible cause of the increase of MAO activity we selected hearts from patients with similar age. A significantly higher activity in both ventricles was found only in IHD, whereas in non-IHD failing hearts MAO activity was similar to that of NF (Figure 1). These data suggest that the increase* of* MAO activity may be related to disease etiology. On the basis of these results further analyses were performed only in IHD failing hearts.

### 3.2. Activity and Expression MAO Isoforms in NF and Failing IHD Ventricles

**Figure 2:**
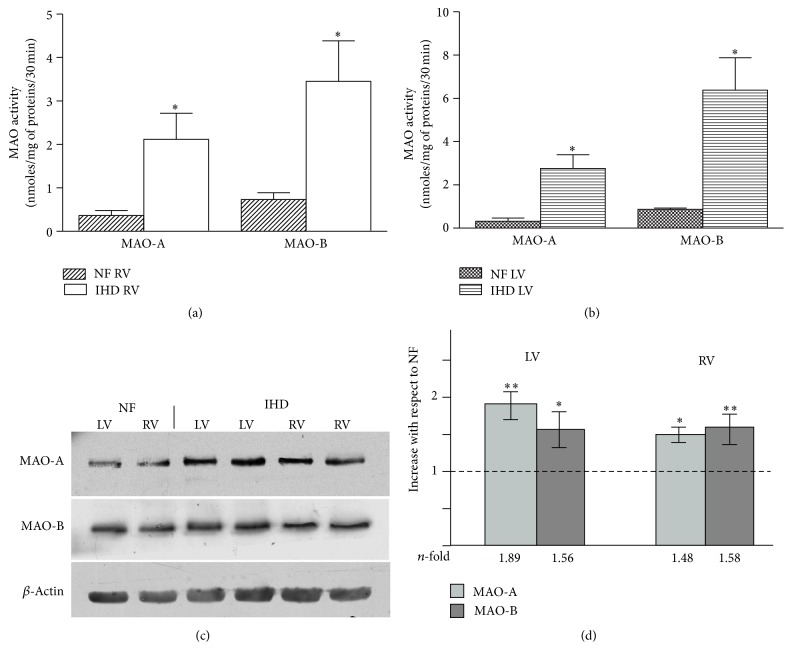
MAO isoform (MAO-A and B) activity (a, b) and expression (c, d) in RV and LV of NF and failing IHD ventricles. Data are expressed as mean ± SEM. ^*∗*^ *p* < 0.05 failing* versus* NF. *n* = 7 NF; *n* = 13 IHD. Representative immunoblots (c) and densitometric quantification (d) of ratio of MAO to *β*-actin protein expression expressed as fold increase of IHD with respect to NF = 1. ^*∗*^ *p* < 0.05 failing* versus* NF; ^*∗∗*^ *p* < 0.01 failing* versus* NF.

To establish the contribution to total MAO activity of either MAO isoform, we measured the activity of MAO-A and MAO-B separately. In NF ventricles, both MAO-A and B isoforms were present with a MAO-A/MAO-B activity ratio of about 1 : 2-3-fold in both ventricles; in IHD ventricles, MAO-A and B activities increased significantly as compared to NF hearts (Figures 2(a) and 2(b)) with a similar increase of the IHD/NF ratio of MAO isoform activity in the respective ventricle (LV: MAO-A 8.40-fold; MAO-B: 7.33-fold; RV: MAO-A 5.92-fold; MAO-B 4.73-fold). The enhanced activities of both MAO isoforms in failing hearts could result from overexpression. Therefore, we measured MAO-A and MAO-B protein expressions by western blotting in the same LV and RV samples in which their activities were evaluated. In accordance with the measured activity, the MAO isoform/*β*-actin ratio determined by densitometric analysis showed that both isoforms were significantly overexpressed in failing ventricles as compared to the values found in NF hearts (NF = 1) (LV: MAO-A 1.89-fold, *p* < 0.01; MAO-B 1.56-fold, *p* < 0.05; RV: MAO-A 1.48-fold, *p* < 0.05; MAO-B 1.58, *p* < 0.01), (Figures 2(c) and 2(d)).

Even though we measured the activity and protein expression of each MAO isoform in human failing hearts, we were unable to distinguish the real contribution to HF of either enzyme. The role of MAO-A in myocardial failure is better described, although the contribution of MAO-B is now emerging as important as MAO-A [20] in this scenario. Therefore, for what the following results are concerned, we considered only the contribution of the MAO-A isoform.

### 3.3. CAT and ALDH-2 Activities

**Figure 3:**
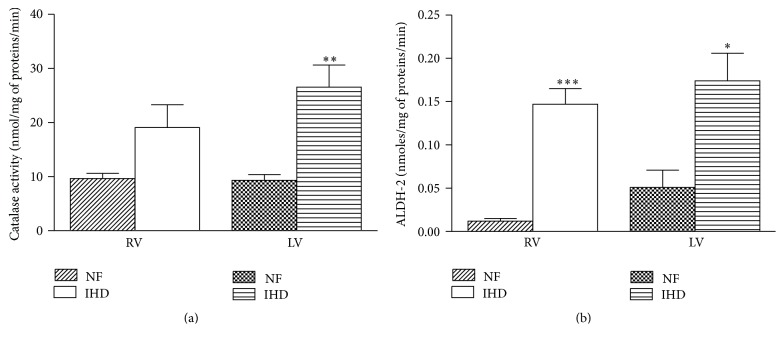
Catalase (a) and ALDH-2 activity (b) in RV and LV of NF and failing IHD ventricles. Data are expressed as mean ± SEM. ^*∗*^ *p* < 0.05 failing* versus* NF LV; ^*∗∗*^ *p* < 0.01 failing* versus* NF LV; ^*∗∗∗*^ *p* < 0.001 failing* versus* NF RV. *n* = 7 NF; *n* = 13 IHD.

With regard to the enzymes involved in MAO catalysis, CAT activity was significantly increased only in the failing LV of IHD hearts (*p* < 0.01) (Figure 3(a)); in this ventricle CAT activity was also significantly correlated to MAO-A activity (*r* = 0.91; *p* < 0.029). Differently from CAT, ALDH-2 activity was significantly higher in both failing ventricles than in NF ones (LV* versus* NF, *p* < 0.05; RV* versus* NF, *p* < 0.001) (Figure 3(b)), whereas it was significantly related to MAO-A activity only in LV (*r* = 0.92; *p* < 0.028).

### 3.4. Oxidative Stress Markers

**Figure 4:**
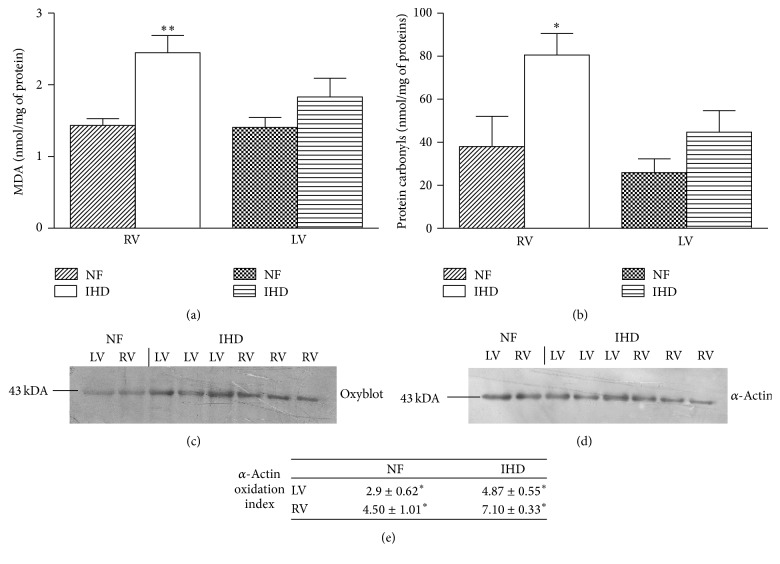
Oxidative stress markers. MDA (a) and protein carbonyls (b) content in RV and LV of NF and failing IHD ventricles. Data are expressed as mean ± SEM. ^*∗*^ *p* < 0.05 failing* versus* NF LV; ^*∗∗*^ *p* < 0.01 failing* versus* NF RV. Actin carbonylation was obtained subjecting derivatized cardiac proteins to SDS-PAGE and immunoblot with anti-DNP antibodies (Oxyblot) (c) followed by stripping and reprobing with anti-actin antibodies (d). Actin oxidation index (e) is given by the ratio between the densitometric values of the bands in the Oxyblot and those of the corresponding bands in the anti-actin immunoblot; ^*∗*^ *p* < 0.05 failing* versus* NF. *n* = 7 NF; *n* = 13 IHD.

Once the activity and expression levels of MAO, CAT, and ALDH-2 in failing RV and LV were assessed, we investigated oxidative stress markers to determine whether the values found were associated with different levels of oxidation products, including MDA and protein carbonyls, typical markers of ROS-mediated attack to lipids and proteins. Our results showed that MDA and protein carbonyls were significantly increased only in failing RV as compared to NF (MDA: IHD* versus* NF, *p* < 0.01; protein carbonyls: IHD* versus* NF, *p* < 0.05) (Figures 4(a) and 4(b)), whereas they were substantially unchanged in LV.

To assess whether total carbonylated proteins measured in failing RV and LV included some myofibrillar proteins critical for contractile performance of heart ventricles, we subjected derivatized proteins to SDS-PAGE coupled to anti-DNP antibodies staining (Oxyblot).  Figure 4(c) shows the presence of a unique band, at 43 kDa, whose migration corresponds to that of *α*-actin and whose identity was confirmed by immunoblot with specific (anti-*α*-actin) antibodies (Figure 4(d)). No band was found at about 37 kDa, corresponding to the Mr of tropomyosin. Interestingly, the ratio between the densitometric value of actin stained with anti-DNP antibodies and that of the corresponding band of *α*-actin, identified by immunoblot (actin oxidation index), was significantly higher than that in NF in both failing RV and LV (LV: 1.68-fold, *p* < 0.05; RV: 1.58-fold, *p* < 0.05), in spite of a nonsignificant change in total carbonylated protein content in failing LV. Finally, the comparison between actin oxidation index observed in failing RV and LV and MAO-A isoform activity showed a positive and significant correlation (RV: *r* = 0.68, *p* = 0.001; LV: *r* = 0.63; *p* = 0.02).

### 3.5. Actin Oxidation Index and MAO-A Isoform Are Related to Functional Heart Parameters

**Figure 5:**
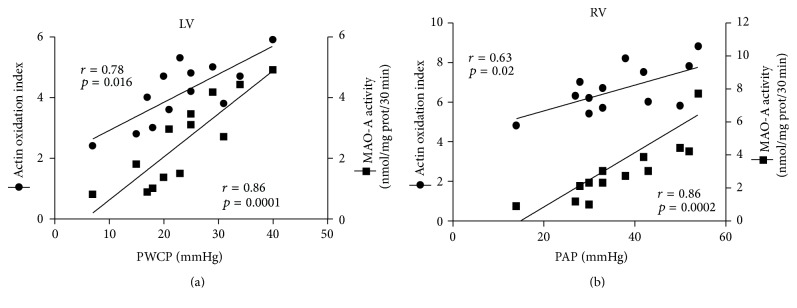
Actin oxidation index and MAO-A activity are correlated to functional parameters in RV and LV of failing IHD ventricles. LV actin oxidation index is significantly correlated with LV MAO-A activity and pulmonary capillary wedge pressure (PCWP) as well as RV actin oxidation index with RV MAO-A activity and pulmonary artery pressure (PAP). *n* = 13 IHD.

It is known that oxidative stress affects heart physiology in normal and ischemic conditions. Accordingly, we tested whether MAO-A isoform activity and actin oxidation index were related to functional heart parameters; among the latter, we considered the pulmonary capillary wedge pressure (PCWP), used to diagnose the severity of left ventricle failure and the pulmonary artery pressure (PAP), an index of right ventricular failure and a negative prognostic marker in HF. As shown in Figures 5(a) and 5(b) all these parameters were positively and significantly correlated in both ventricles, confirming that oxidative stress affects the functional heart parameters.

## 4. Discussion

The present study reports for the first time that human end-stage ischemic heart disease is associated with a significant increase of MAO expression and activity responsible for increased oxidative stress in failing RV and LV. In addition, we found that the adaptive mechanisms needed to cope with the increased oxidative stress are differently activated in failing RV and LV, likely resulting in a greater risk of exposure to oxidative stress damage for the RV than LV failure. Finally, a significant correlation was observed between oxidative modification of actin, a key protein for heart contractility, MAO-A activity, and the two heart functional parameters, PCWP and PAP. This latter finding suggests a mechanistic link among enzyme activation, protein chemical modifications, and functional markers of relevance for cardiac impairment in end-stage HF.

A deleterious role of the MAO-A/ROS pathway has been reported in acute situations such as ischemia-reperfusion, where pharmacological or genetic inactivation of MAO-A prevents cardiac oxidative stress and cardiomyocyte death [9, 12, 37]. However, the importance of the MAO-A/H_2_O_2_ axis in chronic situations such as HF remains poorly understood. Recently, Villeneuve et al. [18] observed that enhanced MAO-A activity* per se* is sufficient to trigger deleterious effects in the heart, particularly in cardiac diseases where MAO-A upregulation was observed [15, 38]. Moreover, Kaludercic et al. [20] showed that, under stress conditions, MAO-B activity contributes to oxidative stress and structural and functional derangements of the heart, establishing a direct relationship between products of MAO activity, oxidative stress, and mitochondrial dysfunction. All these studies were conducted in animal or cellular models. Here we report that, in human heart failure, the expression and activities of MAO isoforms are strongly increased both in RV and in LV from failing hearts in an etiology-dependent manner, in agreement with previous studies [39, 40]. However, we could intriguingly hypothesize that MAO-A and B expression/activity changes are a response to a different accumulation of MAO substrates including NE and 5-HT [41, 42] or to the ischemia-dependent release of humoral factors promoting MAO gene transcription [38, 43]. Accordingly, MAO upregulation could well be an adaptive mechanism yet becoming maladaptive on the long-term.

We also found that two enzymes, CAT and ALDH-2, involved in scavenging of MAO reaction products [3, 44], were differently activated in LV and RV from ischemic hearts. In fact, the activity of both enzymes was significantly enhanced in LV, whereas, in RV samples, only the increase of ALDH-2 was statistically significant. Whatever the case, the increased scavenging capacity of CAT and ALDH-2 in failing hearts may counteract the cytotoxic effects of the products of MAO activity, notably in the LV. In the failing RV, oxidative stress markers, such as MDA and protein carbonylation, were significantly elevated. Thus, the induction of antioxidant and detoxifying capacity in the RV was not sufficient to compensate for the increased oxidative stress, thus exposing the right heart chamber to more severe oxidative damage. These findings agree with our previous study [21] in which we observed that other antioxidant enzyme systems (i.e., Mn-SOD, Gpx) were also differently activated in RV* versus* LV, thus making the RV more sensitive to oxidative stress. Therefore, despite a qualitative similarity of the biochemical mechanisms, generating oxygen and carbonyl reactive species (i.e., MAO activity), the elicited scavenging response was quantitatively different in the RV and LV, suggesting that RV might be less equipped than LV to counter oxidative/carbonyl stress. Accordingly, Littlejohns et al. [45] showed a more prominent change of protein expression profiles in the diseased RV compared to LV. Lipid peroxidation and protein carbonylation are likely to result from severe oxidative stress contributing to dysfunction of heart contractility [46]. We found that the level of total carbonylated proteins was significantly increased only in the RV. However, among sarcomeric myofibrillar proteins, only actin (not tropomyosin) showed a significantly elevated oxidation index and, interestingly, this occurred also in the failing LV. Previous studies demonstrated that actin oxidation can cause formation of protein aggregates as a result of sulfhydryl cross-linking or reaction of one protein radical with an adjacent protein radical, resulting in cross-linking and polymerization. Cross-linking of actin and other contractile proteins could hinder the interactions of thick and thin filaments thus interfering with excitation-contraction coupling and contractile function [47]. Recently, Canton et al. [46] observed that actin carbonylation appears to reflect the oxidative degradation of myofibrillar proteins and that the latter was correlated to contractile impairment in human heart failure.

Interestingly, in our failing ventricles, the level of actin oxidation was significantly related not only to MAO-A activity, but also to PWCP in LV and to PAP in RV, respectively. The correlation between enhanced oxidative stress and PAP was previously observed also in the case of NADPH oxidase, another source of ROS [48]. Our present data suggest a close association between MAO-A-dependent ROS generation, actin oxidation, and ventricular dysfunction, supporting a general conclusion that increased oxidative or/and carbonyl stress, combined with defective antioxidant scavenging system as in the RV, is a hallmark of negative outcome. Indeed, both increased PCWP and RV failures are prognostic markers of progression to overt HF [30–32, 49, 50]. In this scenario, the overactivation of MAO-A in human heart failure represents a novel finding that links oxidative stress to a common alteration and prognostic marker of HF, that is, increased levels of noradrenaline [41], a specific MAO-A substrate. Therefore, the increase of noradrenaline levels and MAO-A activity can represent a synergic, harmful combination, as suggested by the correlation with clinical markers of the disease.

In this study, we focused on MAO-A isoform and its correlation with disease. However, the occurrence of abundant protein level but, above all, the intense activity of MAO-B suggest that MAO-B can also play a pathogenic role in human cardiac diseases. In an experimental model of chronic hemodynamic stress, Kaludercic et al. [20] showed that increased MAO-B activity through ROS and aldehyde production contributes to mitochondrial dysfunction that, in turn, leads to cardiac structural and functional disarrangement; on the contrary, lack or inhibition of MAO-B prevents major cardiac adverse effects. In our opinion, the latter is a very interesting finding and the first evidence that MAO-B plays a role as significant as MAO-A in HF pathogenesis. Accordingly, this new aspect deserves further investigation in human failing hearts.

## 5. Limitations and Conclusions

Despite substantial progress in deciphering individual processes involved in the initiation and gradual progression of HF, our understanding of the molecular mechanisms of HF in humans is undermined by the multifactorial etiology of cardiac dysfunction, by confounding comorbid conditions and also by a lack of appropriate healthy controls. We are aware that our study has significant limitations and needs future extension. Nevertheless, our data provide new information on the molecular/functional mechanisms responsible for the progression towards end-stage heart failure and suggest a new target for the development of more specific therapeutic strategies for heart diseases. Previous evidence shows that protein changes in heart failure from ischemic cardiomyopathy involve mitochondria [40, 51, 52]. A recent study indicates that MAO is a key determinant of redox balance in the human atrial myocardium and a biomarker for postoperative atrial fibrillation, a common complication of heart surgery [53]. Even though the present results point to a possible pathogenic role of MAO in human myocardial failure, in agreement with other studies [53, 54], we cannot exclude the contribution of other ROS sources. An intense cross-talk is likely to exist among different ROS sources, in particular between MAO and NOX; the latter has been convincingly suggested to play a critical role in redox signalling and contractile dysfunction of the heart, as previously reported by our and other studies [1, 48, 52, 55]. It is conceivable that mitochondrial MAO is part of a ROS circuitry that triggers or enhances intracellular NOX activity. Unfortunately, to date, there is a lack of data on the effect of specific NOX inhibitors; consequently, effective clinical therapies targeting NOX remain elusive [1, 56]. On the contrary, MAO inhibitors are available and already used in clinic for treatment of neurodegenerative disorders such as Parkinson's and Alzheimer's diseases [5, 57], and the available information suggests that MAO inhibition is beneficial for treatment of cardiovascular pathologies [58]. In this study we propose these enzymes (and in particular MAO-A) as new pharmacologic targets; moreover, the introduction of a new generation of reversible MAO inhibitors lacking the so called “cheese-effect” [3] makes attractive the idea of exploiting such therapy for clinical use in congestive HF patients.

In conclusion, despite the strong support of a key role of oxidative stress in the pathophysiology of heart failure by experimental studies, the outcome of clinical trials using different antioxidant approaches remains elusive. There is no doubt that some drugs already in use for HF treatment may act indirectly to ameliorate the excess of oxidative stress; accordingly, identifying potential markers and targets for novel HF therapies appears an important research objective.

## Figures and Tables

**Table 1:** Clinical characteristics of the patients.

	NF	IHD	Non-IHD
Total number	7	13	8
Sex (M/F)	5/2	12/1	7/1
Age (range)	52 (45–56)	55 (37–69)	45 (27–63)
LVEF (%)		27 ± 3.8	23 ± 2.5
PAP (mmHg)		38 ± 3.2	29 ± 5.4
PCWP (mmHg)		23 ± 3	21 ± 5.4
RAP (mmHg)		14 ± 4	11 ± 4.4
NYHA class		III-IV	III-IV
Diuretics		7	5
Digoxin		0	2
Antiarrhythmics		3	2
ACE-I		10	5
*β*-blockers		7	5
Nitrates		4	0
Statins		2	0
ANF/rRNA 18S	1.03 ± 0.001	12.4 ± 1.05	12.3 ± 0.98
MCH-*α*/rRNA 18S	1.30 ± 0.03	0.06 ± 0.01	0.04 ± 0.002
MCH-*β*/rRNA 18S	1.35 ± 0.2	0.06 ± 0.02	0.54 ± 0.05

LVEF: left ventricular ejection fraction; ACE-I: angiotensin converting enzyme inhibitors; PCWP: pulmonary capillary wedge pressure; PAP: pulmonary artery pressure; RAP: right atrium pressure; HMGCoA inhibitors (statins); ANF: atrial natriuretic factor; MHC: myosin heavy chain.

## References

[1] Nediani C., Raimondi L., Borchi E., Cerbai E. (2011). Nitric oxide/reactive oxygen species generation and nitroso/redox imbalance in heart failure: from molecular mechanisms to therapeutic implications. *Antioxidants and Redox Signaling*.

[2] Kaludercic N., Mialet-Perez J., Paolocci N., Parini A., Di Lisa F. (2014). Monoamine oxidases as sources of oxidants in the heart. *Journal of Molecular and Cellular Cardiology*.

[3] Kaludercic N., Carpi A., Menabò R., Di Lisa F., Paolocci N. (2011). Monoamine oxidases (MAO) in the pathogenesis of heart failure and ischemia/reperfusion injury. *Biochimica et Biophysica Acta (BBA)—Molecular Cell Research*.

[4] Shih J. C. (2007). Monoamine oxidases: from tissue homogenates to transgenic mice. *Neurochemical Research*.

[5] Youdim M. B. H., Edmondson D., Tipton K. F. (2006). The therapeutic potential of monoamine oxidase inhibitors. *Nature Reviews Neuroscience*.

[6] Berry M. D., Juorio A. V., Paterson I. A. (1994). The functional role of monoamine oxidases A and B in the mammalian central nervous system. *Progress in Neurobiology*.

[7] Finberg J. P. M. (2014). Update on the pharmacology of selective inhibitors of MAO-A and MAO-B: focus on modulation of CNS monoamine neurotransmitter release. *Pharmacology and Therapeutics*.

[8] Naoi M., Riederer P., Maruyama W. (2016). Modulation of monoamine oxidase (MAO) expression in neuropsychiatric disorders: genetic and environmental factors involved in type A MAO expression. *Journal of Neural Transmission*.

[9] Bianchi P., Pimentel D. R., Murphy M. P., Colucci W. S., Parini A. (2005). A new hypertrophic mechanism of serotonin in cardiac myocytes: receptor-independent ROS generation. *The FASEB Journal*.

[10] Villeneuve C., Caudrillier A., Ordener C., Pizzinat N., Parini A., Mialet-Perez J. (2009). Dose-dependent activation of distinct hypertrophic pathways by serotonin in cardiac cells. *American Journal of Physiology—Heart and Circulatory Physiology*.

[11] Sabri A., Hughie H. H., Lucchesi P. A. (2003). Regulation of hypertrophic and apoptotic signaling pathways by reactive oxygen species in cardiac myocytes. *Antioxidants & Redox Signaling*.

[12] Bianchi P., Kunduzova O., Masini E. (2005). Oxidative stress by monoamine oxidase mediates receptor-independent cardiomyocyte apoptosis by serotonin and postischemic myocardial injury. *Circulation*.

[13] Fischer Y., Thomas J., Kamp J. (1995). 5-Hydroxylryptamine stimulates glucose transport in cardiomyocytes via a monoamine oxidase-dependent reaction. *Biochemical Journal*.

[14] Mialet-Perez J., Bianchi P., Kunduzova O., Parini A. (2007). New insights on receptor-dependent and monoamine oxidase-dependent effects of serotonin in the heart. *Journal of Neural Transmission*.

[15] Kaludercic N., Takimoto E., Nagayama T. (2010). Monoamine oxidase A-mediated enhanced catabolism of norepinephrine contributes to adverse remodeling and pump failure in hearts with pressure overload. *Circulation Research*.

[16] Di Lisa F., Kaludercic N., Carpi A., Menabò R., Giorgio M. (2009). Mitochondrial pathways for ROS formation and myocardial injury: the relevance of p66Shc and monoamine oxidase. *Basic Research in Cardiology*.

[17] Carpi A., Menabò R., Kaludercic N., Pelicci P., Di Lisa F., Giorgio M. (2009). The cardioprotective effects elicited by p66^Shc^ ablation demonstrate the crucial role of mitochondrial ROS formation in ischemia/reperfusion injury. *Biochimica et Biophysica Acta—Bioenergetics*.

[18] Villeneuve C., Guilbeau-Frugier C., Sicard P. (2013). P53-PGC-1*α* pathway mediates oxidative mitochondrial damage and cardiomyocyte necrosis induced by monoamine oxidase-a upregulation: role in chronic left ventricular dysfunction in mice. *Antioxidants and Redox Signaling*.

[19] Umbarkar P., Sarojini Singh S., Arkat S., Bodhankar S. L., Lohidasan S., Sitasawad S. L. (2015). Monoamine oxidase-A is an important source of oxidative stress and promotes cardiac dysfunction, apoptosis, and fibrosis in diabetic cardiomyopathy. *Free Radical Biology and Medicine*.

[20] Kaludercic N., Carpi A., Nagayama T. (2014). Monoamine Oxidase b prompts mitochondrial and cardiac dysfunction in pressure overloaded hearts. *Antioxidants and Redox Signaling*.

[21] Borchi E., Bargelli V., Stillitano F. (2010). Enhanced ROS production by NADPH oxidase is correlated to changes in antioxidant enzyme activity in human heart failure. *Biochimica et Biophysica Acta (BBA)—Molecular Basis of Disease*.

[22] Stanley B. A., Sivakumaran V., Shi S. (2011). Thioredoxin reductase-2 is essential for keeping low levels of H_2_O_2_ emission from isolated heart mitochondria. *Journal of Biological Chemistry*.

[23] Budas G. R., Disatnik M.-H., Mochly-Rosen D. (2009). Aldehyde dehydrogenase 2 in cardiac protection: a new therapeutic target?. *Trends in Cardiovascular Medicine*.

[24] Malátková P., Maser E., Wsól V. (2010). Human carbonyl reductases. *Current Drug Metabolism*.

[25] Chen C.-H., Budas G. R., Churchill E. N., Disatnik M.-H., Hurley T. D., Mochly-Rosen D. (2008). Activation of aldehyde dehydrogenase-2 reduces ischemic damage to the heart. *Science*.

[26] Churchill E. N., Disatnik M.-H., Mochly-Rosen D. (2009). Time-dependent and ethanol-induced cardiac protection from ischemia mediated by mitochondrial translocation of *ε*PKC and activation of aldehyde dehydrogenase 2. *Journal of Molecular and Cellular Cardiology*.

[27] Ma H., Guo R., Yu L., Zhang Y., Ren J. (2011). Aldehyde dehydrogenase 2 (ALDH2) rescues myocardial ischaemia/reperfusion injury: role of autophagy paradox and toxic aldehyde. *European Heart Journal*.

[28] Manni M. E., Zazzeri M., Musilli C., Bigagli E., Lodovici M., Raimondi L. (2013). Exposure of cardiomyocytes to angiotensin II induces over-activation of monoamine oxidase type A: implications in heart failure. *European Journal of Pharmacology*.

[29] Santin Y., Sicard P., Yücel Y. (2016). Role and mechanisms of action of aldehydes produced by monoamine oxidase A in cardiomyocyte death and heart failure. *Cardiovascular Research*.

[30] Guglin M., Khan H. (2010). Pulmonary hypertension in heart failure. *Journal of Cardiac Failure*.

[31] Brieke A., DeNofrio D. (2005). Right ventricular dysfunction in chronic dilated cardiomyopathy and heart failure. *Coronary Artery Disease*.

[32] Drazner M. H., Velez-Martinez M., Ayers C. R. (2013). Relationship of right- to left-sided ventricular filling pressures in advanced heart failure. Insights from the escape trial. *Circulation: Heart Failure*.

[33] Raimondi L., Banchelli G., Sgromo L. (2000). Hydrogen peroxide generation by monoamine oxidases in rat white adipocytes: role on cAMP production. *European Journal of Pharmacology*.

[34] Aebi H. (1984). Catalase *in vitro*. *Methods in Enzymology*.

[35] Wenzel P., Hink U., Oelze M. (2007). Role of reduced lipoic acid in the redox regulation of mitochondrial aldehyde dehydrogenase (ALDH-2) activity. Implications for mitochondrial oxidative stress and nitrate tolerance. *The Journal of Biological Chemistry*.

[36] Correa-Salde V., Albesa I. (2009). Reactive oxidant species and oxidation of protein and heamoglobin as biomarkers of susceptibility to stress caused by chloramphenicol. *Biomedicine and Pharmacotherapy*.

[37] Nigmatullina R. R., Kirillova V. V., Jourjikiya R. K. (2009). Disrupted serotonergic and sympathoadrenal systems in patients with chronic heart failure may serve as new therapeutic targets and novel biomarkers to assess severity, progression and response to treatment. *Cardiology*.

[38] Kong S. W., Bodyak N., Yue P. (2005). Genetic expression profiles during physiological and pathological cardiac hypertrophy and heart failure in rats. *Physiological Genomics*.

[39] Roselló-Lletí E., Alonso J., Cortés R. (2012). Cardiac protein changes in ischaemic and dilated cardiomyopathy: a proteomic study of human left ventricular tissue. *Journal of Cellular and Molecular Medicine*.

[40] Li W., Rong R., Zhao S. (2012). Proteomic analysis of metabolic, cytoskeletal and stress response proteins in human heart failure. *Journal of Cellular and Molecular Medicine*.

[41] Schömig A. (1990). Catecholamines in myocardial ischemia. Systemic and cardiac release. *Circulation*.

[42] Shimizu Y., Minatoguchi S., Hashimoto K. (2002). The role of serotonin in ischemic cellular damage and the infarct size-reducing effect of sarpogrelate, a 5-hydroxytryptamine-2 receptor blocker, in rabbit hearts. *Journal of the American College of Cardiology*.

[43] Shih J. C., Chen K. (2004). Regulation of MAO-A and MAO-B gene expression. *Current Medicinal Chemistry*.

[44] Djordjevic J., Jasnic N., Vujovic P. (2012). Distinct and combined effects of acute immobilization and chronic isolation stress on MAO activity and antioxidative protection in the heart of normotensive and spontaneously hypertensive rats. *Journal of Animal Physiology and Animal Nutrition*.

[45] Littlejohns B., Heesom K., Angelini G. D., Suleiman M.-S. (2014). The effect of disease on human cardiac protein expression profiles in paired samples from right and left ventricles. *Clinical Proteomics*.

[46] Canton M., Menazza S., Sheeran F. L., Polverino De Laureto P., Di Lisa F., Pepe S. (2011). Oxidation of myofibrillar proteins in human heart failure. *Journal of the American College of Cardiology*.

[47] Powell S. R., Gurzenda E. M., Wahezi S. E. (2001). Actin is oxidized during myocardial ischemia. *Free Radical Biology and Medicine*.

[48] Nediani C., Borchi E., Giordano C. (2007). NADPH oxidase-dependent redox signaling in human heart failure: relationship between the left and right ventricle. *Journal of Molecular and Cellular Cardiology*.

[49] DeMarco V., Whaley-Connell A., Sowers J., Habibi J., Dellsperger K. (2010). Contribution of oxidative stress to pulmonary arterial hypertension. *World Journal of Cardiology*.

[50] Voelkel N., Quaife R., Leinwand L. (2006). National Heart, Lung and Blood Institute Working Group on Cellular and Molecular Mechanisms of Right Heart Failure Right ventricular function and failure. Report of a National Heart, Lung and Blood Institute Working Group on cellular and molecular mechanisms of right heart failure. *Circulation*.

[51] Roselló-Lletí E., Tarazón E., Barderas M. G. (2015). ATP synthase subunit alpha and LV mass in ischaemic human hearts. *Journal of Cellular and Molecular Medicine*.

[52] Sebastiani M., Giordano C., Nediani C. (2007). Induction of mitochondrial biogenesis is a maladaptive mechanism in mitochondrial cardiomyopathies. *Journal of the American College of Cardiology*.

[53] Anderson E. J., Efird J. T., Davies S. W. (2014). Monoamine oxidase is a major determinant of redox balance in human atrial myocardium and is associated with postoperative atrial fibrillation. *Journal of the American Heart Association*.

[54] Petrak J., Pospisilova J., Sedinova M. (2011). Proteomic and transcriptomic analysis of heart failure due to volume overload in a rat aorto-caval fistula model provides support for new potential therapeutic targets—monoamine oxidase A and transglutaminase 2. *Proteome Science*.

[55] Octavia Y., Brunner-La Rocca H. P., Moens A. L. (2012). NADPH oxidase-dependent oxidative stress in the failing heart: from pathogenic roles to therapeutic approach. *Free Radical Biology and Medicine*.

[56] Casas A. I., Dao V. T.-V., Daiber A. (2015). Reactive oxygen-related diseases: therapeutic targets and emerging clinical indications. *Antioxidants and Redox Signaling*.

[57] Marin D. B., Bierer L. M., Lawlor B. A. (1995). L-deprenyl and physostigmine for the treatment of Alzheimer's disease. *Psychiatry Research*.

[58] Deftereos S. N., Dodou E., Andronis C., Persidis A. (2012). From depression to neurodegeneration and heart failure: re-examining the potential of MAO inhibitors. *Expert Review of Clinical Pharmacology*.

